# Placental MRI and its application to fetal intervention

**DOI:** 10.1002/pd.5526

**Published:** 2019-07-28

**Authors:** Rosalind Aughwane, Emma Ingram, Edward D. Johnstone, Laurent J. Salomon, Anna L. David, Andrew Melbourne

**Affiliations:** ^1^ Institute for Women's Health University College London London UK; ^2^ Division of Developmental Biology & Medicine University of Manchester Manchester UK; ^3^ Hôpital Necker‐Enfants Malades, AP‐HP, EHU PACT and LUMIERE Platform Université Paris Descartes Paris France; ^4^ National Institute for Health Research University College London Hospitals Biomedical Research Centre London UK; ^5^ School of Biomedical Engineering and Imaging Sciences King's College London London UK; ^6^ Medical Physics and Biomedical Engineering University College London London UK

## Abstract

**Objective:**

Magnetic resonance imaging (MRI) of placental invasion has been part of clinical practice for many years. The possibility of being better able to assess placental vascularization and function using MRI has multiple potential applications. This review summarises up‐to‐date research on placental function using different MRI modalities.

**Method:**

We discuss how combinations of these MRI techniques have much to contribute to fetal conditions amenable for therapy such as singletons at high risk for fetal growth restriction (FGR) and monochorionic twin pregnancies for planning surgery and counselling for selective growth restriction and transfusion conditions.

**Results:**

The whole placenta can easily be visualized on MRI, with a clear boundary against the amniotic fluid, and a less clear placental‐uterine boundary. Contrasts such as diffusion weighted imaging, relaxometry, blood oxygenation level dependent MRI and flow and metabolite measurement by dynamic contrast enhanced MRI, arterial spin labeling, or spectroscopic techniques are contributing to our wider understanding of placental function.

**Conclusion:**

The future of placental MRI is exciting, with the increasing availability of multiple contrasts and new models that will boost the capability of MRI to measure oxygen saturation and placental exchange, enabling examination of placental function in complicated pregnancies.

## Abstract 2

What is already known about this topic?

- Placental function is responsible for significant morbidity and mortality in fetal growth restriction and in monochorionic twin pregnancies complicated by selective growth restriction and transfusion conditions.
- Our ability to diagnose placental dysfunction in utero is currently limited, with implications for clinical decision making.
- MRI is capable of imaging the whole human placenta at any gestational age and has been shown to demonstrate differences between normally functioning placentas and those with growth restriction.

What does this study add?

- This review summarises up‐to‐date research on placental function that has been carried out using different MRI modalities.
- We discuss how combinations of these techniques have much to contribute to fetal conditions amenable for therapy such as singletons at high risk for FGR through early recognition, appropriate management, and monitoring response to treatment and monochorionic twin pregnancies for planning surgery and counselling for selective growth restriction and transfusion conditions.

**What is already known about this topic?**

- Placental function is responsible for significant morbidity and mortality in fetal growth restriction and in monochorionic twin pregnancies complicated by selective growth restriction and transfusion conditions.
- Our ability to diagnose placental dysfunction in utero is currently limited, with implications for clinical decision making.
- MRI is capable of imaging the whole human placenta at any gestational age and has been shown to demonstrate differences between normally functioning placentas and those with growth restriction.

**What does this study add?**

- This review summarises up‐to‐date research on placental function that has been carried out using different MRI modalities.
- We discuss how combinations of these techniques have much to contribute to fetal conditions amenable for therapy such as singletons at high risk for FGR through early recognition, appropriate management, and monitoring response to treatment and monochorionic twin pregnancies for planning surgery and counselling for selective growth restriction and transfusion conditions.

## 1 INTRODUCTION

**Figure 1:**
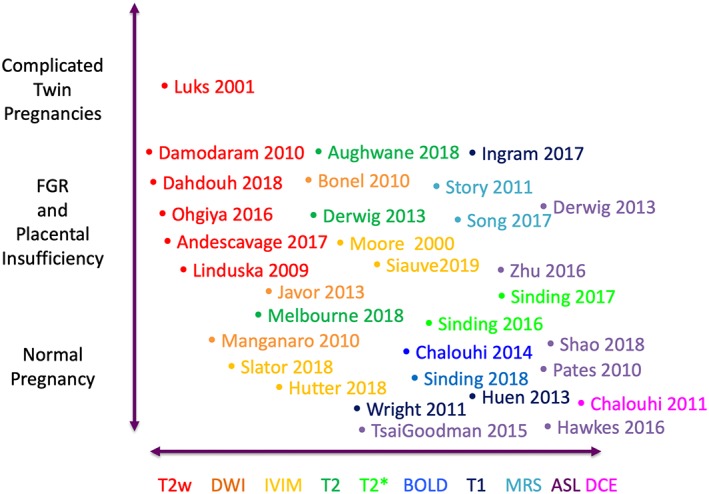
Use of MRI in human placental conditions other than accreta, papers discussed in this review. Abbreviations in text [Colour figure can be viewed at http://wileyonlinelibrary.com]

Magnetic resonance imaging (MRI) of the placenta has been part of clinical practice for many years but is most commonly performed to aid in the diagnosis and management of abnormally adherent placentation. However, there is a growing field investigating imaging of the placenta for other applications (Figure 1). This is down to the technique's ability not only to image structure but also to provide quantitative measures that relate to the tissue properties and function. Several techniques are sensitive to the vascular structure and to properties such as oxygenation and blood flow and thus reveal functional information. Combinations of these techniques have much to contribute to fetal conditions amenable for therapy such as singletons at high risk for fetal growth restriction (FGR) through early recognition, appropriate management, and monitoring response to treatment and monochorionic twin pregnancies for planning surgery and counselling for selective growth restriction and transfusion conditions.

### 1.1 Fetal growth restriction

Placental insufficiency leads to FGR, where a fetus fails to reach their genetic growth potential. Poor fetal nutrition and hypoxia result, with increased risk of cognitive impairment, in cerebral palsy and in lifelong metabolic consequences^1^. The condition is associated with up to two‐thirds of stillbirths in the United Kingdom.^2^, ^3^, ^4^ FGR can be challenging to diagnose as placental function cannot currently be directly measured. Surrogate markers, such as abnormal fetal growth trajectory or abnormal blood flow to the placenta,^5^, ^6^, ^7^, ^8^, ^9^, ^10^, ^11^, ^12^, ^13^, ^14^, ^15^, ^16^, ^17^, ^18^, ^19^, ^20^, ^21^, ^22^, ^23^, ^24^, ^25^, ^26^, ^27^, ^28^, ^29^, ^30^, ^31^, ^32^, ^33^, ^34^, ^35^, ^36^, ^37^, ^38^, ^39^, ^40^, ^41^, ^42^, ^43^, ^44^, ^45^, ^46^, ^47^, ^48^, ^49^, ^50^, ^51^, ^52^, ^53^, ^54^, ^55^, ^56^, ^57^, ^58^, ^59^, ^60^, ^61^, ^62^, ^63^, ^64^, ^65^, ^66^, ^67^, ^68^, ^69^, ^70^, ^71^, ^72^, ^73^, ^74^, ^75^, ^76^, ^77^ are used with varying success. At present, there is no treatment for FGR, or the associated condition pre‐eclampsia; however, trials are exploring several new therapeutic avenues, including sildenafil,^9^ esomeprazole,^10^ metformin,^11^ pravastatin,^12^ and vascular endothelial growth factor maternal gene therapy.^13^, ^14^ Developing new techniques to assess placental function and response to management is therefore essential.^15^, ^16^, ^17^, ^18^

FGR is typically divided into early and late‐onset, most frequently defined as diagnosis before or after 32 weeks of gestation.^5^, ^19^, ^20^ These have relatively different clinical phenotypes, with early‐onset FGR being relatively less common, but with a high incidence of placental pathology, and late‐onset being more common, but with a variety of aetiologies. Clinical challenges in these groups also differ. In early‐onset FGR, the difficulty is in balancing in utero mortality and morbidity against the associated complications of iatrogenic preterm birth,^21^, ^22^, ^23^ whereas in late‐onset FGR, the primary issue is detection and delineation from normal small fetuses. Chronic hypoxia is a critical feature of FGR.^18^, ^24^ It is possible that measurement of fetal or placental oxygen saturation or oxygen exchange may be useful in differentiating the normal small fetus from one with early or late‐onset FGR and might predict outcome.

Placental insufficiency is generally considered to be as a consequence of inadequate spiral artery remodeling from insufficient trophoblast invasion in early pregancy.^25^ The most common abnormal histological finding is patchy placental infarcts.^19^ Lesions relating to hypoxia and therefore suggestive of reduced maternal perfusion are seen more commonly than in normally grown pregnancies. These include syncytiotrophoblast knots, excess cytotrophoblast cells, thickened basement membranes, villous fibrosis, and hypovascular terminal villi, with reduced villous volume, reduced intervillous space, and non‐specific inflammatory lesions.^26^ Understanding this pathophysiology is key to timely diagnosis and management of FGR. Imaging the placenta is therefore important to our understanding and ability to manage FGR.^15^, ^16^, ^17^, ^18^

## 2 COMPLICATED MONOCHORIONIC TWIN PLACENTAS

### 2.1 Twin‐to‐twin transfusion syndrome

In monochorionic twin pregnancies, the two fetuses are intrinsically linked through connections between their circulatory system within the placenta.^27^, ^28^, ^29^, ^30^ Twin‐to‐twin transfusion syndrome (TTTS) is caused by haemodynamic unbalance through these vascular connections,^31^ resulting in one hypovolaemic and one hypervolaemic fetus. If managed conservatively, the overall survival rate for TTTS is around 30%.^32^ Laser surgery to coagulate the anastomosing vessels along the placental equator has been shown to be the most effective management option for severe TTTS.^33^ Increasing information on the location of the vascular equator and the flow mismatch between twins may help clinicians in managing these pregnancies and in planning intervention.

There are limited studies of the villous structure and microcirculation, so placental vascular function is poorly understood. Histological studies have found no difference in histomorphometric variables between shared and nonshared lobules of uncomplicated monochorionic pregnancies.^34^, ^35^ In TTTS however, the donor has reduced average terminal villous diameter, smaller capillaries, reduced vascularization, and larger feto‐maternal diffusion distance, compared with the recipient twin,^34^, ^35^ likely due to the haemodynamic imbalance between the twins.

### 2.2 Selective FGR

Selective FGR (sFGR) is usually regarded as the combination of one twin less than 10th centile for estimated fetal weight (EFW) and a growth discordance between monochorionic twins of greater than 20% to 25% and occurs in 7% to 11% of monochorionic pregnancies.^36^, ^37^, ^38^ It is an important cause of morbidity and mortality.^39^, ^40^ Selective growth restriction provides unique challenges to the obstetrician. Premature delivery comes at the cost of prematurity for the normally grown twin. In some cases, selective reduction of the growth restricted twin is offered in order to optimise the chances for the normally grown fetus. Laser surgery to divide the placentas can also be used, to give both fetuses a chance, whilst protecting the normally grown fetus from harm should the smaller twin die. There is limited information for the clinician on which management option is likely to be the most beneficial for any given situation.

Fetuses with the greater share of the placenta have faster growth velocity than fetuses with the smaller share, unless an arterio‐venous anastomosis is present with net transfusion towards the fetus with the smaller territory which will equalize growth velocities.^29^ Additionally, the presence of an arterio‐arterial anastomosis has been linked to unequal growth in twins with unequal placental share, and absence of an arterio‐arterial anastomosis breaks the association^41^ although this is thought to have a protective association for TTTS. Conversely, an increased proportion of arterio‐venous anastomoses, although rare, is linked with twin anemia polycythemia sequence (TAPS).^42^ Thus, studies suggest a combination of the volume of placental tissue available to each fetus, and the degree and balance of transfusion between them, is responsible for the development of selected growth restriction.^43^

## 3 MAGNETIC RESONANCE IMAGING

### 3.1 Structural Imaging of placenta size and shape

**Figure 2:**
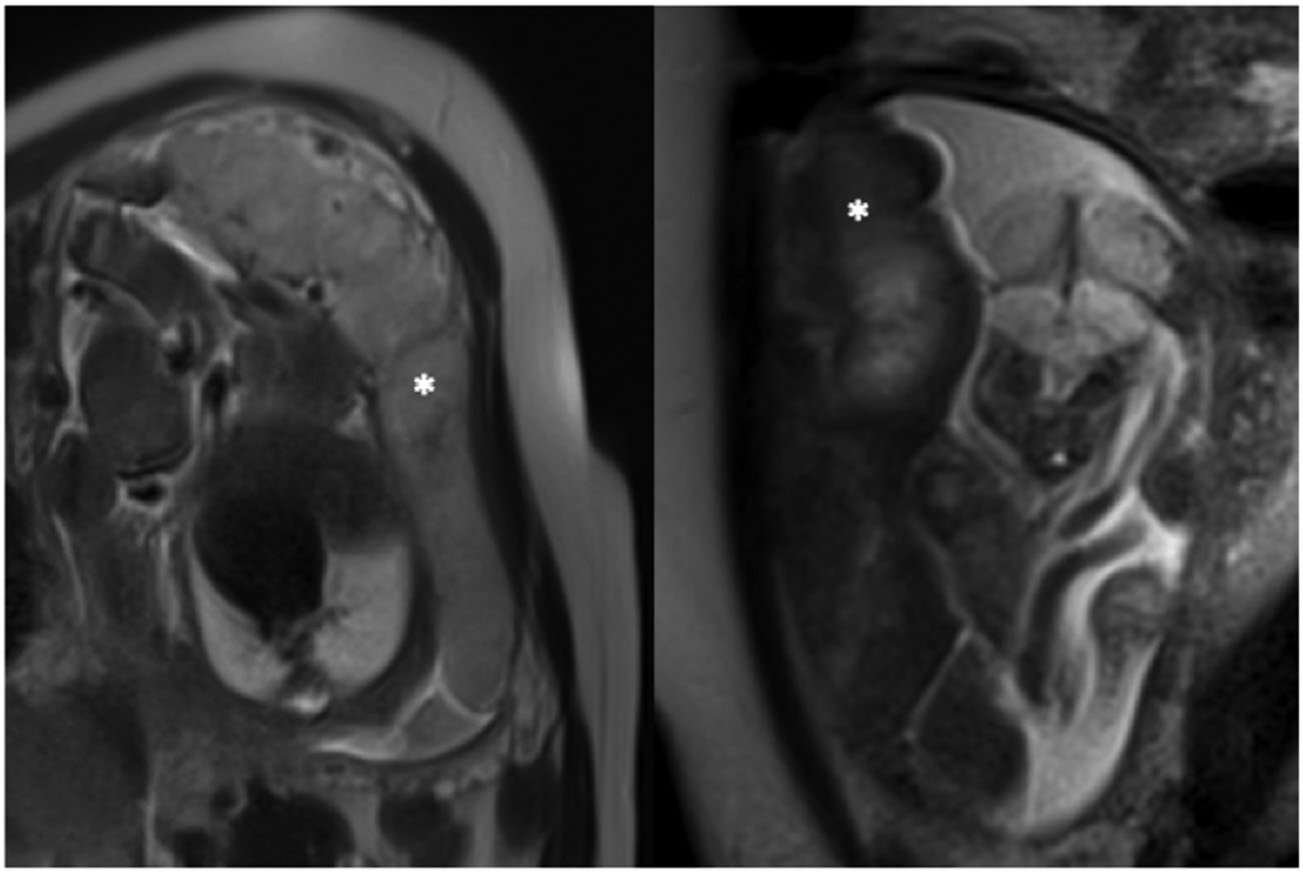
MRI of placenta from a normally grown (left) and FGR (right) fetus. The placenta are marked with white stars. Note the difference in appearance in T2 weighted imaging, with the normal placenta appearing lighter in colour and more homogeneous

The placenta can easily be visualized on MRI, with a clear boundary against the amniotic fluid, and a less clear placental‐uterine boundary (Figure 2). The entire placenta can be imaged at any gestational age, measuring the anatomical size, shape, and vascular properties across the whole organ. MRI is safe in pregnancy.^44^ T2 weighted structural imaging shows a homogenous structure with relatively high T2 signal intensity, giving it a light grey appearance. The T2 value falls in placental insufficiency, giving the placenta a darker appearance, with more heterogeneity, possibly due to areas of infarction and fibrosis.^45^ The placenta is smaller in FGR compared with normally grown controls and has a thickened, globular appearance.^46^ In twin pregnancies, the two cord locations can be seen, and the larger chorionic vessels identified, allowing identification of the vascular equator. Superresolution reconstruction techniques can be used to combine data from 2D stacks acquired in multiple planes into a single 3D volume.^47^ This technique has been applied widely to the fetal brain, and extensions of this technique, although made substantially more complicated by non‐rigid motion, are being used for other abdominal organs.^48^ For placenta size, shape, and thickness estimation, these techniques are likely to represent the best way to acquire data.^46^, ^49^, ^50^, ^51^, ^52^, ^53^ 3D reconstruction of structural MRI data has already been shown to have potential in surgical planning for laser division in TTTS,^54^, ^55^ and as imaging and reconstruction techniques improve is likely to play an increasingly important role.

### 3.2 Diffusion weighted imaging

**Figure 3:**
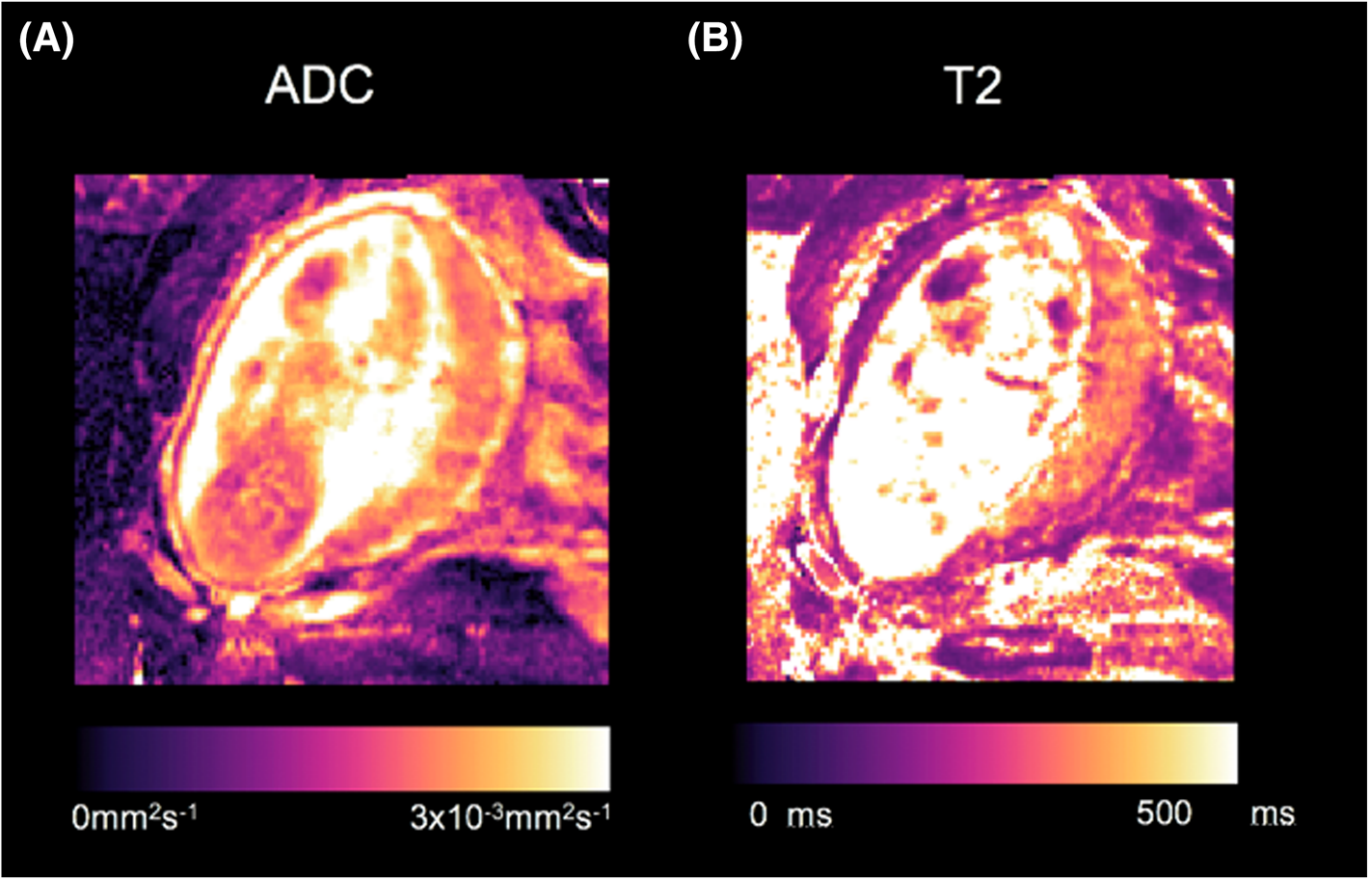
Example of placental single‐compartment ADC and T2 maps generated by linear least‐squares fitting [Colour figure can be viewed at http://wileyonlinelibrary.com]

Diffusion weighted imaging (DWI) is widespread in all areas of medical MRI. The sensitisation of the MRI signal to water movement means that the local tissue structure can be measured by changing the parameters of the diffusion pulses. An apparent diffusion coefficient (ADC) value is calculated for each voxel within an image, and this is displayed as a parametric ADC map (Figure 3A). Voxels with higher ADC values represent a greater degree of water diffusion such as within fluid, whereas voxels with low ADC values represent restricted and hindered diffusion, such as within cellular tissue. The ADC therefore depends on the tissue being imaged, and if pathology is present, and thus, the accuracy and the precision of this value depend on the experimental parameters used.^56^

Several studies have looked at DWI of the growth‐restricted placenta,^57^, ^58^ with placental ADC values being found to be significantly lower in the placentas of FGR pregnancies compared with normal controls and in sFGR.^59^ This suggests the micro‐architectural disturbance in FGR placentas is measurable with MRI.

When DWI is performed in well perfused vascular tissues, the measured signal attenuation at low diffusion sensitisation is due to not only free water diffusion in tissue but also from microcirculation within the capillary network.^60^, ^61^ Intra‐voxel incoherent motion (IVIM)^62^ is the traditional variant of DWI applied to perfused organs. It can be used in the assessment of capillary flow without the need for injecting contrast agents.^63^ As movement of blood within capillaries has no specific orientation and is dependent on the vascular architecture and velocity of the blood it is termed pseudodiffusion. The IVIM model has two compartments, relating to the solid tissue diffusivity and the tissue perfusion, or pseudodiffusivity. The proportion of each signal is given by the perfusion fraction. Naturally, the product of this perfusion fraction with the pseudodiffusivity is a correlate of blood flow. Although the model fitting is prone to noise, several authors have attempted to make fitting more robust.^56^, ^64^, ^65^

Surgically reduced uterine blood flow in animal models can be observed with IVIM imaging,^66^ and in humans, the perfusion fraction has been repeatedly shown to be reduced in placental insufficiency compared with normal placentas.^67^, ^68^, ^69^, ^70^ Caution however should be applied when interpreting quantitative results from single‐contrast MRI which can be confounded by choice of other imaging parameters if not held‐constant; in both the liver and the placenta, quantification of the vascular density is affected by the choice of other image acquisition parameters.^71^, ^72^ Specifically, it has been found that the estimated perfusion fraction in IVIM is dependent on the chosen echo time.^71^ This problem may be overcome using joint models, fitting DWI alongside T2, or T2* relaxation measurements.^69^, ^73^

Diffusion measurements of this type can be enhanced by including directional sensitisation,^65^, ^74^ and this has been used frequently in other organs to reveal the organisation of the tissue structure, especially the brain.^75^ In the placenta, the directional sensitivity might reveal information about the structure of the villous tree and how this changes in pathology such as FGR where insufficient spiral artery remodelling is thought to lead to mechanical damage and immaturity in the fetal villous tree which may reduce the measured diffusion of water. In the human haemomonochorial placenta, the technique may be limited by in vivo motion and pulsatility in contrast to the complicated structural exchange interfaces seen in other mammals. The technique is also, in principle, sensitive to water perfusion. There is now some evidence of directionality in flow in the placenta, particularly near to the chorionic plate,^65^ and this is likely to be associated with net differences in flow properties between chorionic arteries and veins.

Although, to date, most research has been performed investigating singleton growth restriction, in the future, perfusion imaging may be useful to quantify placental perfusion mismatch between twins and the functional volume of placental tissue. This may guide the best location for laser coagulation, ensuring each twin has sufficient functioning tissue to survive, or demonstrate that this is not possible, making selective reduction the safest management option.

### 3.3 Relaxometry

Relaxometry is the measurement of the signal decay rate in MR by both longitudinal (T1) and transverse (T2/T2*) decay. These contrasts can be explored independently by careful choice of pulse sequence. Theoretically, if not practically, these times correspond to independent physical properties of the tissue.

T2 relaxometry is the quantitative measure of hydrogen proton relaxation following excitation with a radio frequency pulse. The rate of relaxation is different for each tissue; tissue has a short T2 relaxation time, whilst blood has a much longer T2 relaxation time^76^, ^77^ (Figure 3B). Tissues with greater all over surface area, whether in the form of cellular membranes or intracellular or extracellular fibrillary macromolecules, tend to have shorter T2 values. In the placenta, T2 relaxation time decreases with increasing gestation,^78^ possibly because of the proportional increase in villous tissue compared with intervillous space, and increasing fibrin volume density.^79^ T2 relaxation times are significantly reduced in placentas from pregnancies complicated by FGR compared with those with appropriate growth, possibly due to increases in fibrosis, necrosis, and infarcts within the placental parenchyma^80^, ^81^, ^82^, ^83^ and reduced fetal oxygen saturation.^24^, ^77^

T2 values are dominated by the level of oxygen saturation^77^, ^84^, ^85^; higher oxygen saturation values result in higher T2 values. MRI may provide a useful indirect measurement for feto‐placental oxygenation since oxygen saturation is difficult to measure directly and invasive methods carry a risk of miscarriage. MRI relaxometry provides a non‐invasive way to measure feto‐placental oxygen levels, which has been partially validated in sheep.^86^, ^87^ Oxygen saturation in the feto‐placental system is typically quite low when compared with healthy adult measures of oxygen saturation and is found to be significantly lower in growth restricted fetuses.^24^, ^88^

Blood oxygenation level dependent (BOLD) MRI is a T2*‐weighted sequence that is able to detect changes in the proportion of deoxyhaemoglobin and hence reflects tissue oxygen saturation. This technique has found much use for mapping brain function where spatial patterns are used to understand functional networks^89^, ^90^ but is increasingly finding other applications outside of the brain for its ability, in combination with other flow measurements, to measure oxygen extraction and thus efficiency.^91^ However, the interpretation of the placental BOLD signal is complex, with signal changes dependent on other factors including blood flow, blood volume fraction, and haematocrit.^81^, ^92^, ^93^

**Figure 4:**
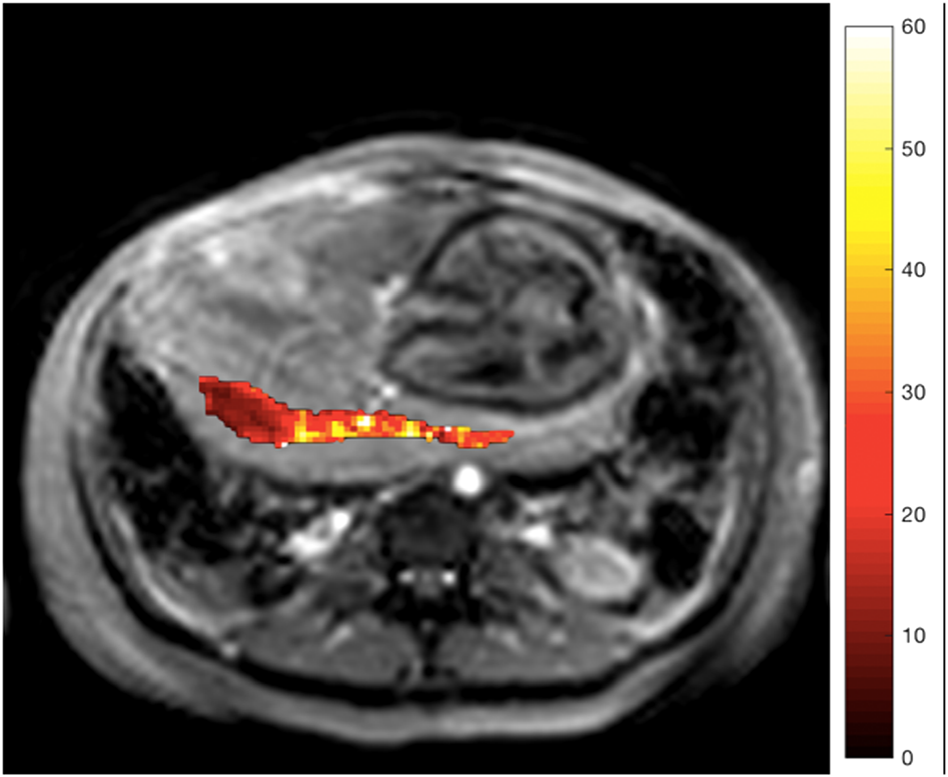
T2 weighted structural image of axial slice through maternal abdomen, demonstrating uterine cavity, fetus, and placenta. Superimposed R2* map of the placental ROI (s^−1^) [Colour figure can be viewed at http://wileyonlinelibrary.com]

BOLD and T2* measurements are often conflated in the literature. The T2* value cannot be directly related to tissue oxygenation as tissue morphology also affects the T2* value, with a reduction in T2* of the placenta with increasing gestation^94^ (Figure 4). This gestational relationship may be related to the histological maturation of the placenta and the decrease in placental oxygenation as pregnancy advances.^95^ During a maternal oxygen‐challenge (hyperoxia), the difference in the absolute T2* value (ΔT2*) signals the change in placental oxygenation independent of baseline conditions, thus demonstrating tissue oxygen saturation. Changes in BOLD signal with controlled hyperoxia and in FGR have been demonstrated in the placenta and other fetal organs.^96^, ^97^ However, a difference in ΔT2* has not been demonstrated in cases of placental dysfunction related to FGR to date despite conflicting animal data.^81^, ^98^, ^99^, ^100^

In T1‐weighted oxygen‐enhanced (OE) MRI,^98^, ^101^, ^102^ the signal change related to the maternal oxygen‐challenge reflects changes in tissue pO2, due to the paramagnetic properties of dissolved oxygen. Compared with BOLD, the absolute signal change seen in OE MRI declines with gestational age and is significantly lower in pregnancies with FGR.^98^, ^101^ This is thought to support the theory of a relative placental hypoxia in FGR related to placental dysfunction, as more of the dissolved oxygen becomes bound to deoxyhaemoglobin, and hence, less becomes dissolved within the tissue.

The potential to estimate fetal oxygen saturations non‐invasively has obvious potential in the management of singleton and twin growth restriction. It could inform on response to treatment, and also on timing of delivery and might relate to placental function, allowing assessment of each lobule of the placenta. The dependence of T2 on haematocrit may also be useful in assessment of TTTS, and if TAPS is suspected.

### 3.4 Multicompartment multicontrast models

Conventional T1, T2, and T2* relaxometry are limited having no physiological correlate outside of MRI and an often unknown or intractable dependence on physiological properties of interest such as blood flow, saturation, haematocrit, or cellular composition. Pure tissue regions such as fluid can sometimes be used to infer properties directly,^103^ but these are more often the exception rather than the rule. Most regions of tissue within an imaging voxel will be mixed, particularly in the heterogenous placenta where fetal blood, maternal blood, and tissue are present within any given voxel. Using joint acquisition protocols, it can be possible to separate the signal contributions from different tissue types.^71^, ^72^, ^73^ This approach does allow physiological properties of the tissue to be inferred, providing the window for potential histological, complementary, or invasive validation methods.

**Figure 5:**
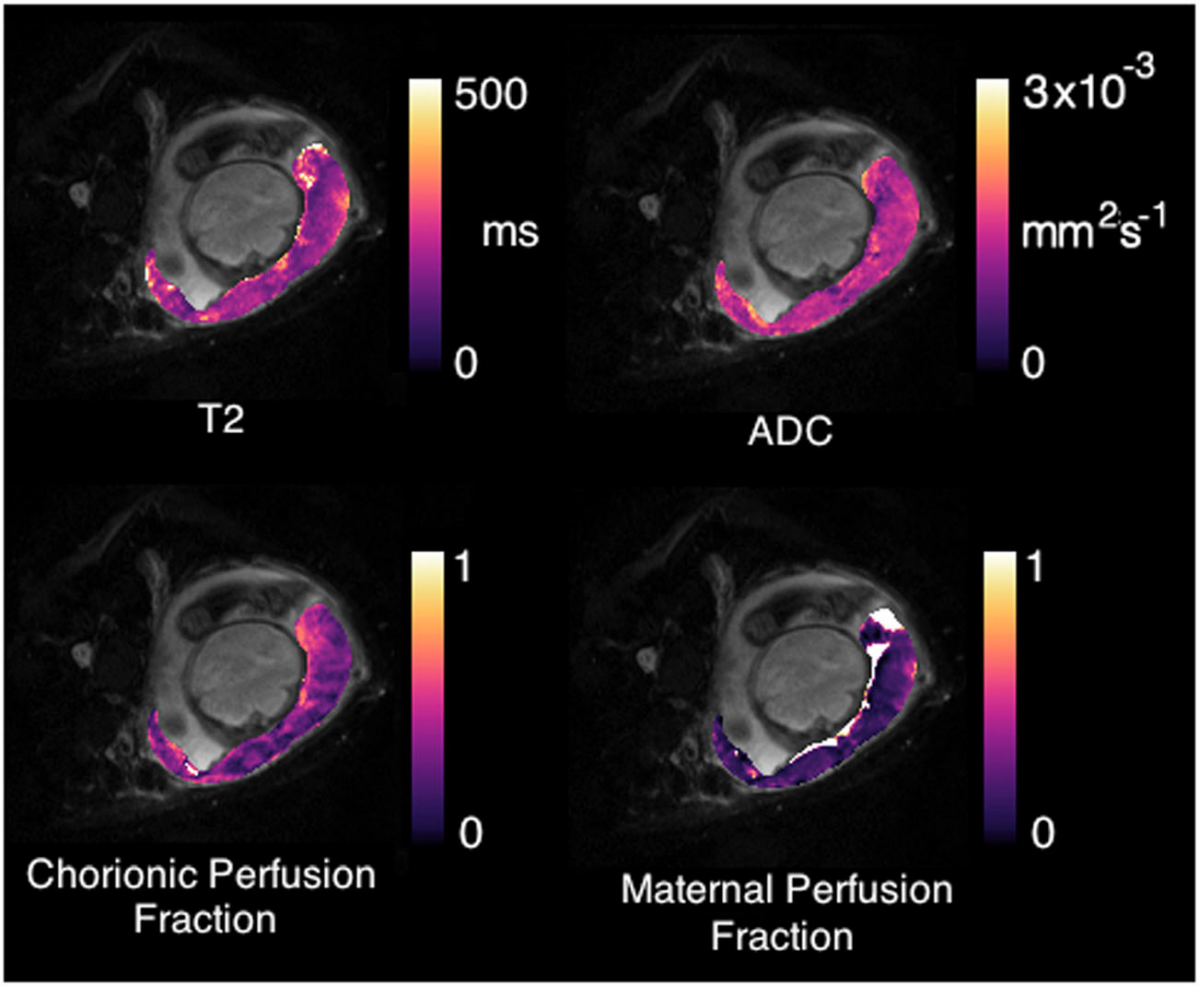
Physiological model‐fitting of the placenta.^72^ Parametric maps can be produced corresponding to fetal and maternal perfusion fractions (bottom row) simultaneously to conventional ADC and T2 maps (top row) [Colour figure can be viewed at http://wileyonlinelibrary.com]

Multicompartment multicontrast models of the type used in DWI can also be generated. The first multicompartment placental specific model is DECIDE,^72^ which separates the different T2 values of fetal and maternal blood from the background tissue compartment (Figure 5). Doing this results in a mechanism, under certain assumptions, to measure the fetal blood oxygen saturation. This model can also be applied to combined DWI and T2* data. Multicontrast models of this type represent a paradigm shift in the use of MRI for FGR, giving a non‐invasive measurement of placental function.^72^, ^73^ Models such as these carry their own assumptions about the physics and physiology of the signal generation process and so researchers should be aware of the limitations of each model for specific pathologies. In general, they carry the same goal of scanner‐independence as for single‐compartment models of T2 or diffusivity, in principle allowing the combination and comparison of data between sites and populations but additionally allowing further validation work because of their physical motivation.

### 3.5 MRI flow and metabolic measurement

**Figure 6:**
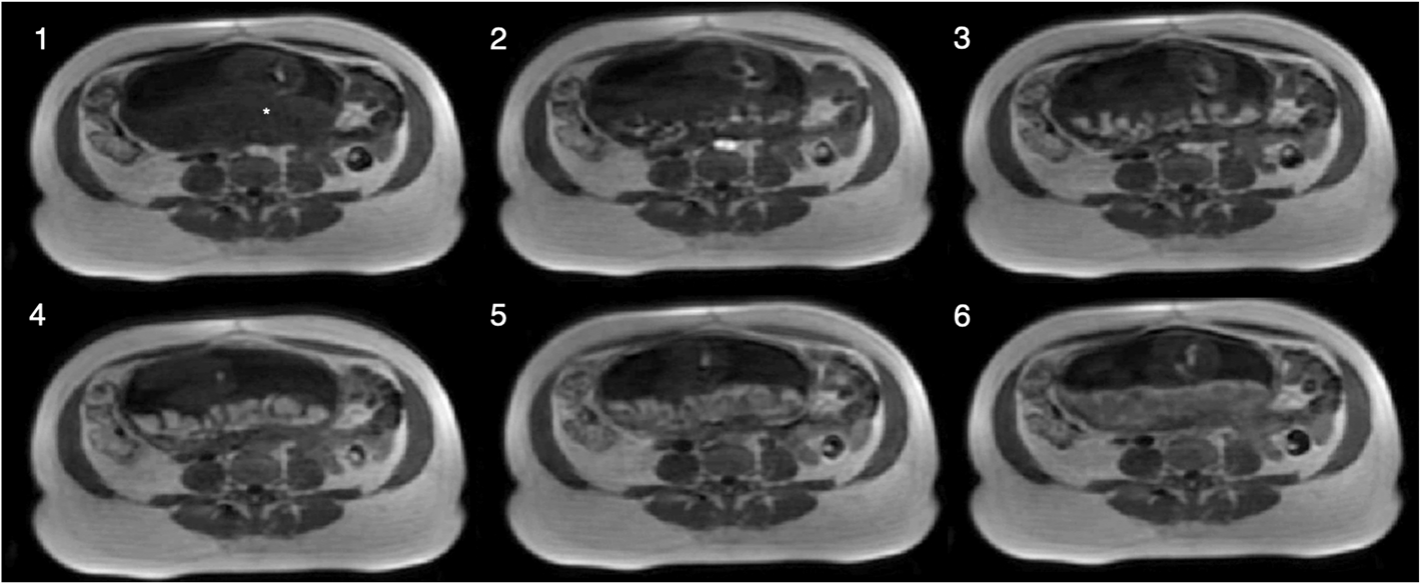
Dynamic enhancement of the placenta with DCE‐MRI. Baseline image (1), arrival and wash‐in (2‐4), wash‐out (5‐6)

A key area of MR research is the measurement of the vascular properties of a tissue. The gold‐standard technique for this uses an injected para‐magnetic contrast agent that makes it unsuitable for fetal and maternal clinical MRI except in the most extreme circumstances.^104^, ^105^ Dynamic contrast enhanced (DCE) MRI^80^, ^87^, ^106^ does have the capability to reveal the pharmaocokinetics of the placenta including the input of blood to the uterus and placenta and the exchange of contrast agent into the trophoblast and across to the fetus (Figure 6). Common models describe the delivery of contrast to the maternal side of the placenta and the transfer of contrast agent into the fetal blood pool, thus having the potential to improve our understanding of how these processes are affected in different pathologies.^105^, ^107^, ^108^ However, the decision to use contrast to image complex pregnancies is challenging.

Flow can be measured with phase contrast MRI, an imaging technique that encodes the blood flow velocity in large arteries, typically of several millimetres in diameter, directly into the MR imaging data. In combination with knowledge of the vessel area, this gives a quantitative estimate of blood flow.^109^, ^110^ Due to the readily available use of Doppler ultrasound, there is little work in this area.^111^, ^112^

Arterial spin labeling is a further imaging technique that magnetically labels blood water to visualise larger arterial vessels and blood perfusion.^67^, ^113^, ^114^ Arterial blood water is magnetically labelled just below the region of interest using a radiofrequency inversion pulse. This magnetised tracer flows into the slice of interest, reducing the total tissue magnetisation, and consequently reducing the MR signal and image intensity. The difference between a labelled and unlabelled control image provides a measure of perfusion.^115^ ASL is exquisitely sensitive to motion and can be relatively time consuming to acquire due to the low average signal. However, its key strength is the ability to acquire multiple different labels with differing postlabel delays or different velocity encodings, thus revealing much about the dynamic perfusion of the placenta. A comparison of IVIM and ASL to assess placental perfusion in the second trimester in normal and FGR pregnancies showed a significant reduction in basal plate ASL signal between normally grown and FGR pregnancies. Basal plate, central placental, and whole placental IVIM vascular density was also different between normally grown and FGR pregnancies.^67^ As with IVIM, this technique could be useful in monitoring response to treatment in FGR placentas and also perfusion differences in twin pregnancies. The benefit of this technique is that it is a more direct measurement of perfusion; however, it is challenging to apply in practice.

Placenta metabolites can be measured in principle using MRI via proton magnetic resonance spectroscopy which has been investigated in the placenta. However, high acquisition failure rates and difficulty in interpreting the signal mean this is a relatively immature technique within the placenta.^116^, ^117^

Lastly, although to the best of our knowledge, it has not yet been tested in humans, hyperpolarised MRI represents a unique way to assess the placental barrier and its metabolic behaviour and permeability.^118^ The use of different hyperpolarized metabolites could reveal a range of information on different pathways and pathology far beyond that obtained from pharmacokinetic studies of Gadolinium chelates or other heavy contrast molecules.

## 4 CONCLUSION

The ability of MRI to detect changes in placentas of severely growth restricted fetuses with abnormal Doppler's is well established.^70^, ^78^, ^80^, ^94^ However, the ability of MRI to measure placental function more broadly has yet to be fully realized or investigated (Table 1). With further development, MRI is likely to increase our understanding of abnormal placental function, improve diagnostic accuracy, and help guide intervention and monitor response. The advances currently being made in the examination of placentas from pregnancies affected by growth restriction will find application in wider conditions such as complicated twin pregnancies, invasive placentation, chorioangioma, caesarean scar pregnancies, and the function of other fetal organs.

**Table 1:** Future applications of MRI in placental conditions amenable to therapy

Technique	MRI Signal Sensitivity	Future Applications
T2 weighted	Structural features, fluid boundaries, volumetrics	Placental share in complicated twins, cord insertions, chorionic vessel mapping, computer assisted surgical planning
DWI	Diffusivity, microarchitecture, fluid not specific to oxygenation/flow.	Microvascular structural differences in FGR/PET/sFGR
IVIM	Diffusivity, microvasculature, fluid, perfusion. Chorionic flow. Non‐specific to oxygenation	Functional share in complicated twins. Flow changes in FGR. Post‐intervention redistribution + outcome prediction.
T2weighted	Sensitive to oxygenation, tissue compartments	Changes in fetal oxygenation functional redundancy and capacity
T2*	Sensitive to oxygenation, tissue compartments	Changes in fetal oxygenation, functional redundancy and capacity
BOLD	Sensitive to functional change in oxygenation	Changes in function, and tissue redundancy and capacity over time
T1	Sensitive to oxygenation	Maternal blood flow changes in FGR. Redistribution postlaser TTTS
MRS and metabolic	Transfer rates, tissue maturation	Therapeutic changes in transfer and exchange
ASL	Sensitive to flow and perfusion	Maternal blood flow changes in FGR. Redistribution postlaser TTTS
DCE	Sensitive to flow and transfer rate	Changes in maternal flow and transfer kinetics.

One of the limitations to the practical use of placental MRI is the relative rarity of some of the conditions being investigated. This can make it difficult to establish studies with sufficient numbers to fully investigate new imaging techniques and hence make recommendations about clinical practice. Enhanced coordination of studies between centres and the sharing of clinical and technical expertise alongside imaging data are essential when investigating these conditions^119^ and will help to establish the most useful imaging technologies for each pathology. This will speed up the pace of future feto‐placental research for conditions that for the ubiquity of pregnancy remain quite rare but have lifelong impact.

The future of placental MRI is exciting; the use of multiple contrasts and new models to boost the capability of MRI to measure oxygen saturation^72^ and placental exchange^105^, ^118^ will enhance the understanding of placental function in complicated pregnancies.

## FUNDING SOURCES

This research was supported by the Wellcome Trust (210182/Z/18/Z and Wellcome Trust/EPSRC NS/A000027/1). The funders had no direction in the study design, data collection, data analysis, manuscript preparation, or publication decision.

## CONFLICT OF INTEREST

We have no conflicts of interest to report.

## Data Availability

Data sharing is not applicable to this article as no new data were created or analysed in this study.
